# Estimating the influencing factors for T1b/T2 gallbladder cancer on survival and surgical approaches selection

**DOI:** 10.1002/cam4.6297

**Published:** 2023-06-27

**Authors:** Jiasheng Cao, Jiafei Yan, Jiahao Hu, Bin Zhang, Win Topatana, Shijie Li, Tianen Chen, Sarun Jeungpanich, Ziyi Lu, Shuyou Peng, Xiujun Cai, Mingyu Chen

**Affiliations:** ^1^ Department of General Surgery Sir Run‐Run Shaw Hospital, Zhejiang University Hangzhou Zhejiang Province China; ^2^ Zhejiang University School of Medicine Zhejiang University Hangzhou Zhejiang Province China; ^3^ Department of General Surgery the Second Affiliated Hospital, Zhejiang University Hangzhou Zhejiang Province China

**Keywords:** gallbladder cancer, influencing factors, surgical approaches, survival, time to treatment

## Abstract

**Background:**

The influencing factors, especially time to treatment (TTT), for T1b/T2 gallbladder cancer (GBC) patients remain unknown. We aimed to identify the influencing factors on survival and surgical approaches selection for T1b/T2 GBC.

**Methods:**

We retrospectively screened GBC patients between January 2011 and August 2018 from our hospital. Clinical variables, including patient characteristics, TTT, overall survival (OS), disease‐free survival (DFS), surgery‐related outcomes, and surgical approaches were collected.

**Results:**

A total of 114 T1b/T2 GBC patients who underwent radical resection were included. Based on the median TTT of 7.5 days, the study cohort was divided into short TTT group (TTT ≤7 days, *n* = 57) and long TTT group (TTT >7 days, *n* = 57). Referrals were identified as the primary factor prolonging TTT (*p* < 0.001). There was no significance in OS (*p* = 0.790), DFS (*p* = 0.580), and surgery‐related outcomes (all *p* > 0.05) between both groups. Decreased referrals (*p* = 0.005), fewer positive lymph nodes (LNs; *p* = 0.004), and well tumor differentiation (*p* = 0.004) were all associated with better OS, while fewer positive LNs (*p* = 0.049) were associated with better DFS. Subgroup analyses revealed no significant difference in survival between patients undergoing laparoscopic or open approach in different TTT groups (all *p* > 0.05). And secondary subgroup analyses found no significance in survival and surgery‐related outcomes between different TTT groups of incidental GBC patients (all *p* > 0.05).

**Conclusions:**

Positive LNs and tumor differentiation were prognostic factors for T1b/T2 GBC survival. Referrals associating with poor OS would delay TTT, while the prolonged TTT would not impact survival, surgery‐related outcomes, and surgical approaches decisions in T1b/T2 GBC patients.

## Graphical abstract

1. The delayed TTT was driven by preoperative referrals in T1b/T2 GBC patients.
2. The delayed TTT would not impact survival and surgery‐related outcomes in T1b/T2 GBC patients.
3. Surgeons should make surgical approaches decisions for T1b/T2 GBC patients based on their surgical skills rather than TTT.

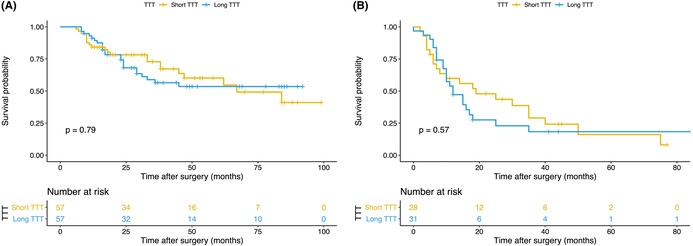

## 1 INTRODUCTION

Gallbladder cancer (GBC), which accounts for 80%–95% of biliary tract cancers, is the sixth most common type of gastrointestinal malignancies worldwide.^1^, ^2^ GBC is a highly fatal disease, with a median overall survival (OS) of 6 months and a 5‐year survival rate of 5%.^3^, ^4^, ^5^ According to the 8th American Joint Committee on Cancer (AJCC) Staging Manual and National Comprehensive Cancer Network (NCCN) Clinical Practice Guidelines (Hepatobiliary Cancers, Version 2.2021), radical/extended resection is the primary approach for T1b/T2 GBC patients,^6^, ^7^ and adjuvant therapy is recommended postoperatively.^8^, ^9^ Although a variety of treatment scenario have emerged, including targeted therapy,^10^ immunotherapy,^11^, ^12^ and several second‐line treatment such as mFOLFOX,^13^ timely surgery is potentially beneficial for advanced GBC patients.

Influencing factors, especially time to treatment (TTT), may be affected by patients' preoperative conditions.^14^ TTT has been implicated in numerous clinical studies for various cancer types, but no definitive conclusion has been reached. A previous cohort study evaluated the delayed TTT for breast, lung, colon, and prostate cancers, and researchers discovered that all of these cancers benefited from a short TTT.^15^ In contrast, a timely TTT may not affect survival outcomes of patients with early‐stage gastric cancer^16^ and advanced pancreatic cancer patients.^17^ Prolonged TTT accompanied by tumor progression may affect surgery‐related outcomes such as operation time, intraoperative blood loss, and postoperative hospital length of stay, which were indicators of operative difficulty.^18^, ^19^ However, the mechanisms underlying TTT and its effect on outcomes in T1b/T2 GBC patients remain unknown. Moreover, the laparoscopic approach was noninferior to the open approach in terms of short‐ and long‐term outcomes for T1b/T2 GBC patients without considering TTT.^20^ Despite the development of laparoscopic surgery, surgeons would hesitate to choose between laparoscopic and open surgical approaches due to potential tumor progression with increased technical difficulty after long TTT.^21^

The purpose of this study was to determine the influencing factors, especially TTT, on survival and surgery‐related outcomes in T1b/T2 GBC patients. Additionally, we sought to provide surgeons with preoperative guidance on T1b/T2 GBC patients that would be used to inform surgical approaches selection.

## 2 METHODS

### 2.1 Study design and population

The electronic medical records of consecutive GBC patients between January 2011 and August 2018 were screened for eligible patients with the limited inclusion biases. The inclusion criteria for the study were as follows: (1) Patients aged from 18 to 80; (2) preoperative suspicion of GBC based on imaging examination (ultrasonography, computed tomography, magnetic resonance imaging, or positron emission tomography‐computed tomography); (3) pathological confirmation of T1b or T2 GBC after radical resection according to the 8th AJCC Staging Manual; (4) no prior medical history of malignancy; and (5) available postoperative follow‐up (≥3 months). Additionally, GBC patients who (1) lacked sufficient baseline data and study outcomes; (2) had a positive resection margin (R1/R2); (3) underwent palliative surgery; and (4) did not undergo hepatectomy (wedge resection or Segment IVb/V resection) or lymph nodes (LNs) dissection were excluded. The retrospective study was reviewed and approved by the Institutional Review Board (IRB) of our hospital (20220305‐31), with patient consent waived.

### 2.2 Patient characteristics

Clinical variables of baseline characteristics, which included age, sex, body mass index (BMI), smoking history, comorbidities, preoperative imaging examination or intraoperative histopathology for GBC, referrals, preoperative jaundice, gallstones, preoperative carbohydrate antigen 19‐9 (CA19‐9), carcinoembryonic antigen (CEA), tumor size, T stage, GBC surgical approaches, total harvested LNs, positive LNs, tumor differentiation, and adjuvant therapy information, were collected. Adjuvant treatment consisted of chemotherapy, radiotherapy, chemoradiotherapy, immunotherapy, targeted therapy, supportive care, and traditional medicine within 3 months postoperatively. The time interval between the initial suspicion of GBC based on imaging examination or histopathology diagnosis of GBC and the date of radical resection was defined as TTT.

### 2.3 Primary and secondary outcomes

The primary outcomes examined in the study were survival, including OS and disease‐free survival (DFS). OS and DFS were defined as the time intervals from radical resection until death and tumor recurrence or the last follow‐up, respectively. Based on the regular telephone follow‐up (every 3 months) or the latest electronic outpatient medical records, the patients' follow‐up data would be obtained. All GBC patients were followed postoperatively until January 2020. The secondary outcomes included operation time, intraoperative blood loss, and postoperative hospital length of stay.

### 2.4 Subgroup analyses of surgical approaches based on different TTT

To examine the effect of TTT on the surgical approaches selection in T1b/T2 GBC, both the short TTT group (median TTT) and the long TTT group (> median TTT) were divided into laparoscopic and open groups, respectively. Subgroup analyses were conducted to compare the clinical variables of baseline characteristics, survival outcomes, and surgery‐related outcomes of different surgical approaches based on TTT.

### 2.5 Secondary subgroup analyses of incidental GBC (IGBC) based on different TTT

IGBC patients are often going through surgery because of gallstone disease or incidental finding of gallbladder polypoid lesions. For IGBC patients, we also defined the TTT as the time duration from the date of tissue histopathology to the date of radical resection, which was similar to GBC patients above. Secondary subgroup analyses were conducted to explore the effect of TTT on survival and surgery‐related outcomes in T1b/T2 IGBC.

### 2.6 Statistical analysis

Categorical variables were presented as frequency and percentage, and they were assessed by the *χ* ^2^ test between two groups. Meanwhile, continuous variables were described using the median and range or means and standard deviations, which were compared using the Wilcoxon rank‐sum test or Student's *t*‐test. Prognostic factors for OS and DFS in GBC were identified by univariable (*p* < 0.1) and multivariable (*p* < 0.05) logistic regression analyses. Kaplan–Meier survival analyses, using the “survival” package in R Studio (Version 4.0.4, R Foundation for Statistical Computing, Vienna, Austria), were conducted to analyze OS and DFS between two groups. Statistical analyses were performed by SPSS (Version 20.0, IBM SPSS) and GraphPad Prism (Version 8.4.0, GraphPad Software Inc.). A *p* value of <0.05 was considered significant.

## 3 RESULTS

### 3.1 Patient selection

**FIGURE 1:**
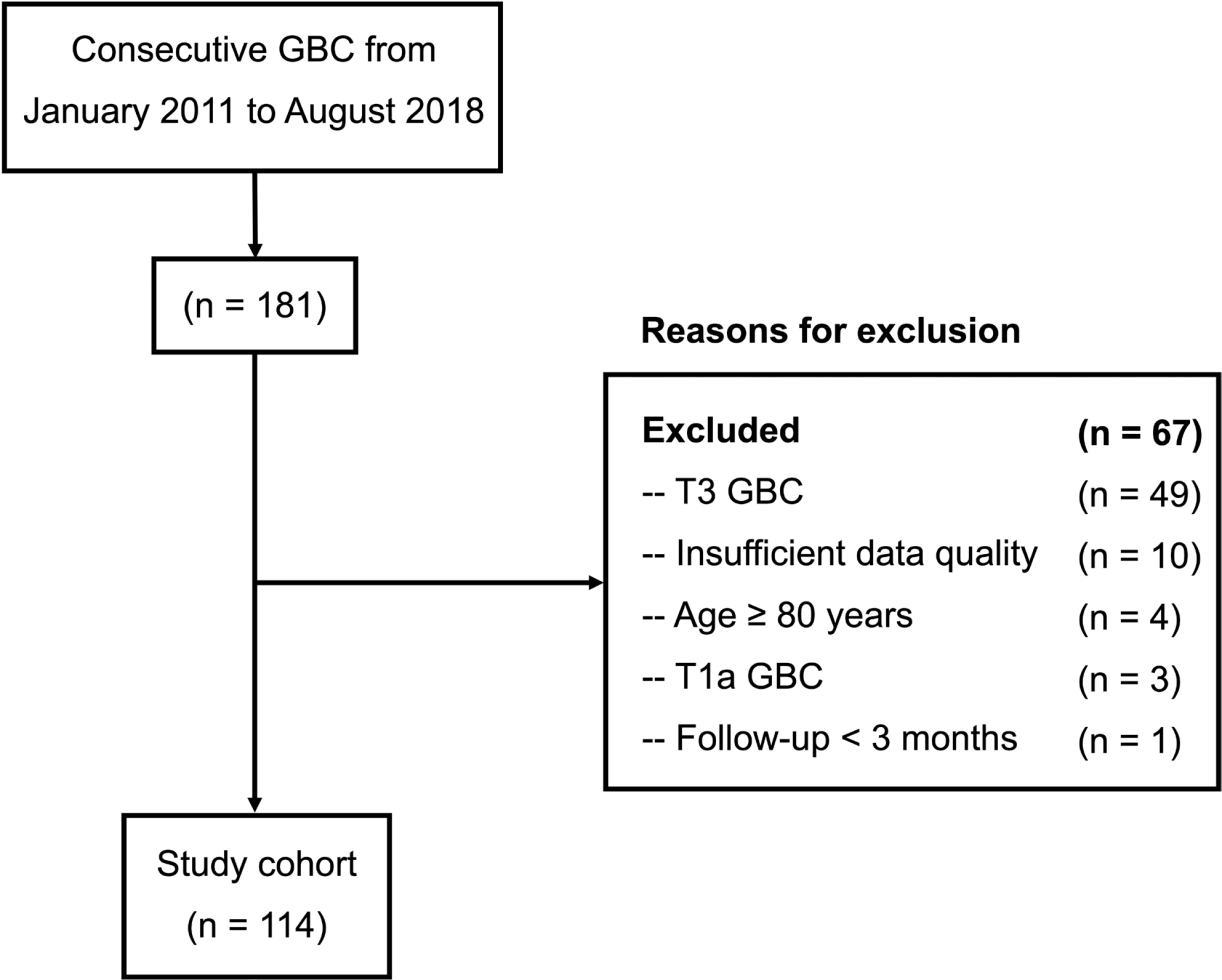
Flow diagram of the study cohort meeting the inclusion and exclusion criteria. GBC, Gallbladder cancer. T stage was based on the 8th American Joint Committee on Cancer Staging Manual.

We retrospectively evaluated 181 consecutive GBC patients at our hospital between January 2011 and August 2018. The study excluded 49 patients with T3 tumors, 10 with insufficient data, 4 who were over the age of 80, 3 with T1a tumors, and 1 with a follow‐up less than 3 months. The remaining 114 T1b/T2b GBC patients undergoing radical resection were included (Figure 1).

### 3.2 Patient characteristics

The The patient characteristics of the GBC patients included in this study are summarized in Table 1. The median age was 62 years [range: 39–79], and 82 (71.9%) patients were female. Histopathology accounted for 43.0% of the examination for GBC, while ultrasonography [23 (20.2%)] and computed tomography [31 (27.2%)] were the most frequently used imaging examinations. Additionally, over one‐third of patients were referred to our hospital [44 (38.6%)] and were diagnosed with gallstones [47 (41.2%)]. Preoperative CA19‐9 and CEA were assessed within the normal range in the majority of patients [85 (74.6%) and 99 (86.8%), respectively], but tumor size and differentiation varied significantly among GBC patients, with tumors of 1–3 cm [57 (50.0%)] and well differentiation [54 (47.4%)] being the most prevalent. Notably, the laparoscopic approach [53 (46.5%)] was performed nearly as much as the open approach [61 (53.5%)]. And most GBC [108 (94.7%)] patients received wedge resection.

**TABLE 1:** Patient characteristics of the study cohort.

Variables	Study cohort (*n* = 114) *n* (%) or median [range]	Short TTT (≤7 days) (*n* = 57) *n* (%) or median [range]	Long TTT (>7 days) (*n* = 57) *n* (%) or median [range]	*p* value
Age (years)	62 [39–79]	64 [45–78]	60 [39–79]	0.740
Sex				
Male	32 (28.1)	15 (26.3)	17 (29.8)	0.677
Female	82 (71.9)	42 (73.7)	40 (70.2)	
BMI ≥25 kg/m^2^	41 (36)	22 (38.6)	19 (33.3)	0.558
Smoking history	7 (6.1)	3 (5.3)	4 (7.0)	0.696
Comorbidities				
Hypertension	38 (33.3)	19 (33.3)	19 (33.3)	0.346
Diabetes mellitus	6 (5.3)	2 (3.5)	4 (7.0)	
Chronic hepatitis B	2 (1.8)	1 (1.8)	1 (1.8)	
History of cerebrovascular accident	2 (1.8)	0 (0)	2 (3.5)	
Coronary artery disease	2 (1.8)	1 (1.8)	1 (1.8)	
Chronic obstructive pulmonary disease	1 (0.9)	0 (0)	1 (1.8)	
Preoperative suspicion or intraoperative diagnosis				
Ultrasonography	23 (20.2)	11 (19.3)	12 (21.1)	0.603
CT	31 (27.2)	12 (21.1)	19 (33.3)	
MRI	9 (7.9)	5 (8.8)	4 (7.0)	
PET‐CT	2 (1.8)	1 (1.8)	1 (1.8)	
Histopathology	49 (43.0)	28 (49.1)	21 (36.8)	
Referrals	44 (38.6)	10 (17.5)	34 (59.6)	**<0.001** *
Preoperative jaundice	0 (0)	0 (0)	0 (0)	NA
Gallbladder stones	47 (41.2)	26 (45.6)	21 (36.8)	0.341
Preoperative CA19‐9 (≤37 U/mL)	85 (74.6)	45 (78.9)	40 (70.2)	0.282
Preoperative CEA (≤5 ng/mL)	99 (86.8)	52 (91.2)	47 (82.5)	0.166
Tumor size (cm)				
≤1	22 (19.3)	15 (26.3)	7 (12.3)	0.151
1–3	57 (50.0)	27 (47.4)	30 (52.6)	
>3	35 (30.7)	15 (26.3)	20 (35.1)	
T stage				
T1b	8 (7.0)	4 (7.0)	4 (7.0)	>0.999
T2	106 (93.0)	53 (93.0)	53 (93.0)	
Surgical approach				
Laparoscopic approach	53 (46.5)	27 (47.4)	26 (45.6)	0.851
Open approach	61 (53.5)	30 (52.6)	31 (54.4)	
Hepatectomy type				
Wedge resection	108 (94.7)	54 (94.7)	54 (94.7)	1.00
Segment IVb/V resection	6 (5.3)	3 (5.3)	3 (5.3)	
Total harvested LNs	7 [1–42]	6 [1–20]	8 [1–42]	0.392
Positive LNs	0 [0–6]	0 [0–5]	0 [0–6]	0.202
Tumor differentiation				
Well	54 (47.4)	28 (49.1)	26 (45.6)	0.930
Moderate	23 (20.2)	11 (19.3)	12 (21.1)	
Poor	37 (32.5)	18 (31.6)	19 (33.3)	
Postoperative adjuvant treatment				
Chemotherapy	11 (9.6)	6 (10.5)	5 (8.8)	0.563
Radiotherapy	2 (1.8)	0 (0)	2 (3.5)	
Chemoradiotherapy	21 (18.4)	12 (21.1)	9 (15.8)	
Immunotherapy	0 (0)	0 (0)	0 (0)	
Targeted therapy	1 (0.9)	0 (0)	1 (1.8)	
Supportive care	76 (66.7)	37 (64.9)	39 (68.4)	
Traditional medicine	3 (2.6)	2 (3.5)	1 (1.8)	

*Note*: T stage was based on the 8th American Joint Committee on Cancer Staging Manual.

Abbreviations: BMI, body mass index; CA19‐9, carbohydrate antigen 19‐9; CEA, carcinoembryonic antigen; CT, computed tomography; LNs, lymph nodes; MRI, magnetic resonance imaging; NA, not available; PET‐CT, positron emission tomography‐computed tomography; TTT, Time to treatment.

* *p* < 0.05.

### 3.3 Drivers of prolonged TTT

**FIGURE 2:**
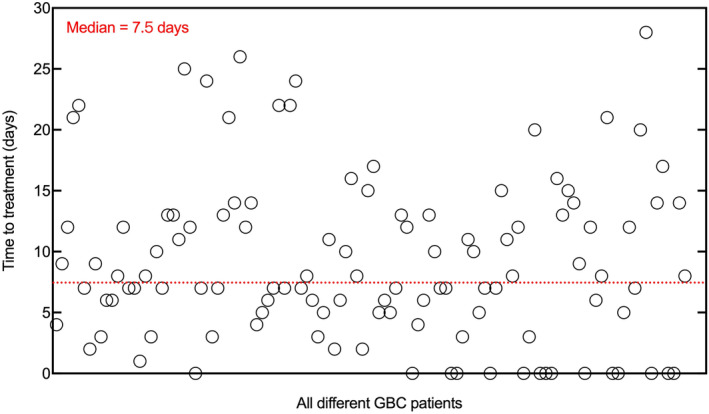
Time to treatment of all different GBC patients. GBC, Gallbladder cancer.

Based on the median TTT of 7.5 days [range: 0–28 days] (Figure 2), the study cohort was divided into the short TTT group (TTT ≤7 days) and the long TTT group (TTT >7 days). Based on the Wilcoxon rank‐sum test, there were 57 patients in the short TTT group with a median of 5 days [range: 0–7 days] and 57 patients in the long TTT group with a median of 13 days [range: 8–28 days] (*p* < 0.001). The patient characteristics of both two groups are summarized in Table 1 with an adequate balance for most variables (*p* > 0.05). Notably, referrals were identified as the most important driver of prolonged TTT (*p* < 0.001). While there was no significant difference among other potential factors related to TTT, such as BMI, smoking history, comorbidities, preoperative imaging examination and intraoperative histopathology for GBC, gallstones, preoperative CA19‐9, and CEA (all *P* > 0.05).

### 3.4 Association of TTT with survival outcomes and surgery‐related outcomes

**FIGURE 3:**
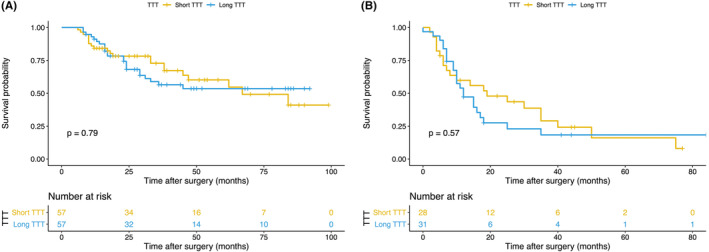
Comparisons of survival outcomes of T1b/T2 GBC patients between two groups. The difference between (A) overall survival (HR, 0.923; 95% CI, 0.511–1.668; *p* = 0.790) and (B) disease‐free survival (HR, 0.844; 95% CI, 0.462–1.540; *p* = 0.580) between short TTT group and long TTT group. GBC, Gallbladder cancer; HR, hazard ratio; CI, confidence interval; TTT, time to treatment.

**FIGURE 4:**
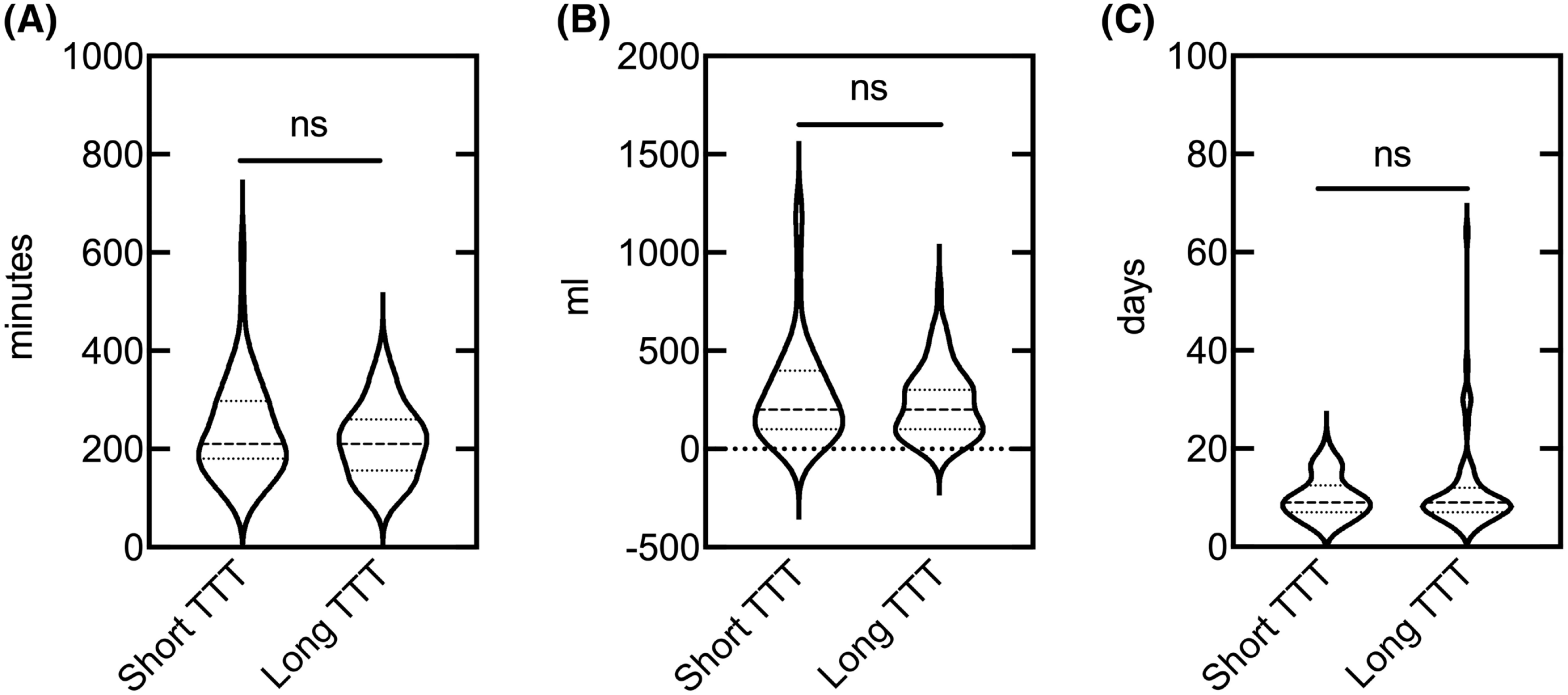
Comparisons of surgery‐related outcomes of T1b/T2 GBC patients between two groups. The difference between (A) operation time (*p* = 0.503), (B) intraoperative blood loss (*p* = 0.756), and (C) postoperative hospital length of stay (*p* = 0.651) between short TTT group and long TTT group. GBC, Gallbladder cancer; TTT, time to treatment; NS, not significant.

Patients with short TTT had no significant benefits over those with long TTT in OS (hazard ratio [HR], 0.923; 95% confidence interval [CI], 0.511–1.668; *p* = 0.790, Figure 3A) and DFS (HR, 0.844; 95% CI, 0.462–1.540; *p* = 0.580, Figure 3B). As for surgery‐related outcomes, no significance of operation time (*p* = 0.503), intraoperative blood loss (*p* = 0.756), and postoperative hospital length of stay (*p* = 0.651) was found between the short TTT group and the long TTT group (Figure 4).

### 3.5 Other influencing factors on OS and DFS

The prognostic factors for OS in GBC patients were identified using univariate and/or multivariate analyses, including referrals (HR, 2.429; 95% CI, 1.301–4.536; *p* = 0.005), positive LNs (HR, 1.321; 95% CI, 1.095–1.595; *p* = 0.004), and tumor differentiation (HR, 1.787; 95% CI, 1.202–2.658; *p* = 0.004; Table 2). Meanwhile, we also identified positive LNs (HR, 1.253; 95% CI, 1.001–1.568; *p* = 0.049) as the sole prognostic factor for DFS (Table 3). Notably, TTT was not considered as a potential risk factor for OS (HR, 0.923; 95% CI, 0.511–1.668; *p* = 0.790, Table 2) and DFS (HR, 0.844; 95% CI, 0.462–1.540; *p* = 0.580, Table 3) in T1b/T2 GBC patients using the Cox proportional hazards model, further confirming the absence of an association between TTT and survival benefits.

**TABLE 2:** Prognostic factors for overall survival in GBC patients based on univariable and multivariable analyses.

Variables	Univariable analysis			Multivariable analysis		
	HR	95% CI	*p* value	HR	95% CI	*p* value
Age (years)	1.009	0.975–1.044	0.620			
Sex	0.691	0.364–1.312	0.259			
Male						
Female						
BMI ≥25 kg/m^2^	1.221	0.647–2.305	0.538			
Smoking history	1.382	0.493–3.871	0.538			
Comorbidities	1.109	0.599–2.051	0.743			
Preoperative suspicion or intraoperative diagnosis	1.162	0.633–2.134	0.628			
Imaging examination (Ultrasonography/CT/MRI/PET‐CT)						
Histopathology						
Referrals	2.593	1.426–4.716	**0.002** *	2.429	1.301–4.536	**0.005** *
TTT	0.923	0.511–1.668	0.790			
Short TTT (≤7 days)						
Long TTT (>7 days)						
Gallbladder stones	1.277	0.684–2.384	0.442			
Preoperative CA19‐9 (≤37 U/mL)	0.567	0.307–1.048	**0.070** *	0.564	0.271–1.175	0.126
Preoperative CEA (≤5 ng/mL)	0.457	0.225–0.925	**0.030** *	0.883	0.390–2.003	0.766
Tumor size (cm)	NA	NA	0.803			
≤1	0.774	0.362–1.654	0.508			
1–3	0.836	0.370–1.886	0.665			
>3	Reference	Reference	Reference			
T stage	0.045	0–10.835	0.267			
T1b						
T2						
Surgical approach	1.572	0.866–2.855	0.137			
Laparoscopic approach						
Open approach						
Hepatectomy type	4.923	1.861–13.027	**0.001** *	1.837	0.644–5.236	0.255
Wedge resection						
Segment IVb/V resection						
Total harvested LNs	1.009	0.966–1.054	0.692			
Positive LNs	1.490	1.264–1.755	**<0.001** *	1.321	1.095–1.595	**0.004** *
Tumor differentiation	1.897	1.340–2.684	**<0.001** *	1.787	1.202–2.658	**0.004** *
Postoperative adjuvant treatment	NA	NA	0.184			

*Note*: T stage was based on the 8th American Joint Committee on Cancer Staging Manual.

Abbreviations: BMI, body mass index; CA19‐9, carbohydrate antigen 19‐9; CEA, carcinoembryonic antigen; CI, confidence interval; CT, computed tomography; GBC, Gallbladder cancer; HR, hazard ratio; LNs, lymph nodes; MRI, magnetic resonance imaging; NA, not available; PET‐CT, positron emission tomography‐computed tomography; TTT, time to treatment.

* *p* < 0.1.

**TABLE 3:** Prognostic factors for disease‐free survival in GBC patients based on univariable and multivariable analyses.

Variables	Univariable analysis			Multivariable analysis		
	HR	95% CI	*p* value	HR	95% CI	*p* value
Age (years)	1.029	0.993–1.067	0.120			
Sex	1.156	0.608–2.200	0.658			
Male						
Female						
BMI ≥25 kg/m^2^	0.978	0.516–1.851	0.944			
Smoking history	1.725	0.613–4.853	0.302			
Comorbidities	1.071	0.575–1.994	0.829			
Preoperative suspicion or intraoperative diagnosis	1.382	0.752–2.541	0.298			
Imaging examination (Ultrasonography/CT/MRI/PET‐CT)						
Histopathology						
Referrals	1.520	0.836–2.763	0.170			
TTT	0.844	0.462–1.540	0.580			
Short TTT (≤7 days)						
Long TTT (>7 days)						
Gallbladder stones	1.343	0.713–2.527	0.361			
Preoperative CA19‐9 (≤37 U/mL)	0.988	0.528–1.848	0.971			
Preoperative CEA (≤5 ng/mL)	0.931	0.456–1.900	0.844			
Tumor size (cm)	–	–	0.680			
≤1	0.782	0.364–1.679	0.528			
1–3	0.693	0.303–1.585	0.385			
>3	Reference	Reference	Reference			
T stage	NA	NA	NA			
T1b						
T2						
Surgical approach	1.225	0.677–2.218	0.503			
Laparoscopic approach						
Open approach						
Hepatectomy type	2.662	1.027–6.900	**0.044** *	1.515	0.488 – 4.701	0.472
Wedge resection						
Segment IVb/V resection						
Total harvested LNs	1.020	0.981–1.060	0.322			
Positive LNs	1.305	1.077–1.580	**0.007** *	1.253	1.001–1.568	**0.049** *
Tumor differentiation	1.339	0.938–1.910	0.108			
Postoperative adjuvant treatment	0.985	0.812–1.195	0.875			

*Note*: T stage was based on the 8th American Joint Committee on Cancer Staging Manual.

Abbreviations: BMI, body mass index; CA19‐9, carbohydrate antigen 19‐9; CEA, carcinoembryonic antigen; CI, confidence interval; CT, computed tomography; GBC, Gallbladder cancer; HR, hazard ratio; LNs, lymph nodes; MRI, magnetic resonance imaging; NA, not available; PET‐CT, positron emission tomography‐computed tomography; TTT, time to treatment.

* *p* < 0.1.

### 3.6 Impact of TTT on selection of surgical approaches

Based on our previous study, the laparoscopic approach was noninferior to the open approach for T1b/T2 GBC patients without considering TTT.^20^ Subgroup analyses were further performed to reveal the impact of TTT on the selection of surgical approaches. The clinical characteristics of patients undergoing laparoscopic or open approach for T1b/T2 GBC in the short TTT group (all *p* > 0.05) and long TTT group (most *P* > 0.05) were comparable (Table S1). In terms of survival outcomes, there was no significant difference in OS or DFS between patients undergoing laparoscopic and open approaches, regardless of whether they were in the short or long TTT group (all *p* > 0.05; Figure S1). Additionally, an adequate statistical balance was observed in surgery‐related outcomes for both two surgical approaches regardless of TTT, including operation time, intraoperative blood loss, and postoperative hospital length of stay (all *p* > 0.05; Figure S2).

### 3.7 Impact of TTT on T1b/T2 IGBC

IGBC patients (*n* = 49) were selected from the study cohort (Table S2). The median and range of TTT of IGBC was 7 [0–26] days after tissue histopathology. IGBC patients had several reasons for referral, including abdominal pain [33 (67.3%)], abdominal distension [1 (2.0%)], examination for gallbladder stones [7 (14.3%)], and examination for gallbladder polypoid lesions [8 (16.3%)]. Most IGBC patients had abdominal pain [32 (65.3%)], followed by no symptom and [15 (30.6%)] and abdominal distension [2 (4.1%)]. There were 5 (10.2%) T1b IGBC patients and 44 (89.8%) T2 IGBC patients receiving radical surgery, respectively. Based on the TTT of the study cohort, there was almost no significant difference between IGBC patients of short TTT and long TTT groups (Table S3). Notably, for IGBC patients, referrals were the driver of prolonged TTT (*p* = 0.002); meanwhile, surgeons preferred laparoscopic approaches (*p* = 0.026). And no significance was found in OS (*p* = 0.97), DFS (*p* = 0.70), operation time (*p* = 0.322), intraoperative blood loss (*p* = 0.176), and postoperative hospital length of stay (*p* = 0.549) between two groups in IGBC (Figures S3 and S4).

## 4 DISCUSSION

The study demonstrated that fewer positive LNs and well tumor differentiation were influencing factors for T1b/T2 GBC survival. Referrals associating with poor OS would delay TTT, while the prolonged TTT would not impact survival (OS and DFS), surgery‐related outcomes (operation time, intraoperative blood loss, and postoperative hospital length of stay), and surgical approaches decisions in T1b/T2 GBC patients treated with radical resection. Notably, surgeons would not always consider TTT when deciding between laparoscopic and open approaches.

The primary factor that contributed to our study cohort's prolonged TTT was referrals from external medical centers, which have been previously reported in a variety of gastrointestinal cancer types, including colorectal and pancreatic cancers.^17^, ^22^ Prolonged TTT was associated with referrals, an increased risk of mortality, and a worse survival outcome for stage I colorectal cancers.^22^ Fortunately, there was no evidence of an adverse prognosis for patients with resected pancreas cancer treated at high‐volume cancer centers.^23^ In the current study, although GBC patients who were referred from local hospitals to high‐volume centers may have a decreased OS rather than DFS, the absence of a strong association between TTT and survival outcomes was reassuring. It has been reported that the prognosis of GBC depended on the volume of the medical center.^24^ Thus, whenever possible, patients with T1b/T2 GBC should be referred to a specialized high‐volume medical center regardless of the possibility of a TTT delay.

Both preoperative imaging examinations and intraoperative histopathology for GBC did not significantly prolong the TTT of GBC patients and were not associated with an increased risk of OS or DFS. In clinical practice, ultrasonography, computed tomography, and magnetic resonance imaging were considered the most frequently used imaging modalities for GBC.^25^ Among imaging modalities, magnetic resonance imaging showed the best diagnostic value, followed by ultrasonography and computed tomography.^26^ Additionally, surgeons relied on the emerging positron emission tomography‐computed tomography for more precise GBC staging.^27^ Despite the fact that intraoperative histopathology was expensive and would add significantly to pathologists' workload, it was considered standard practice for detecting the presence of GBC.^28^ Nevertheless, the diagnostic modalities and the time required for confirmation varied among GBC patients, and they did not significantly prolong the TTT or negatively impact the survival outcomes. Furthermore, surgeons should select the optimal diagnostic modalities based on the local medical conditions rather than the time required for diagnosis. And other patient‐related factors such as BMI, smoking history, comorbidities, gallstones, preoperative CA19‐9, and CEA were available that would not take long time to analyze or obtain, and these factors would not affect TTT potentially.

The number of positive LNs and tumor differentiation were identified as potential influencing factors for survival outcomes in GBC. Several high‐performance models were developed and validated to predict long‐term outcomes in GBC patients based on clinicopathological factors such as positive LNs and tumor differentiation. Positive LNs may affect the prognosis of GBC after surgery through tumor immune responses.^29^, ^30^ Specifically, in GBC patients with fewer positive LNs, increased dendritic cell infiltration resulted in a more pronounced host lymphocytic response and a weaker LNs micro‐metastasis, thereby improving survival.^31^ Similarly, tumor differentiation, which may reflect biological characteristics, was found to be associated with GBC tumor aggressiveness. In well‐differentiated GBC patients, a lower cellular density of the glandular structure was observed, as was less pericholecystic infiltration and regional LN enlargement, all of which contributed to a better prognosis.^32^ In our study, the short TTT and long TTT (within 28 days) groups did not differ in terms of positive LNs and tumor differentiation, implying that surgeons could guide T1b/T2 GBC patients regarding the optimal timing of radical resection.

The prolonged TTT may influence the surgical approaches chosen. In the long TTT group, tumor progression was associated with increasing operative difficulty, affecting surgeons' choice of laparoscopic or open approach. Despite the fact that our previous study focused on the impacts of surgical approaches on T1b/T2 GBC and demonstrated that laparoscopic surgery was comparable to open surgery in terms of short‐ and long‐term outcomes,^20^ the effect of TTT was not considered. Differently, we included TTT and aimed to reveal the influence of TTT on selection of surgical approaches in the study. Interestingly, no significant survival or surgery‐related outcomes were observed. The possible reason for this was that patients treated after 2011 were included in the study, and the milestone year of 2011 saw significant advancements in laparoscopic treatment of GBC,^33^ whether in hepatectomy strategy^34^, ^35^ or LNs dissection management.^36^ Therefore, surgeons should choose optimal surgical approaches depending on their surgical skills and hospital levels instead of TTT.

Numerous studies have been conducted to determine the impact of TTT as a potential influencing factor on surgical survival outcomes in the field of digestive surgery. Ethun et al.^37^ performed a multicenter study to evaluate the association or TTT of salvage surgery with OS for incidental GBC. Although a prolonged TTT of 4–8 weeks was identified as the optimal time interval, the study excluded preoperative suspicion of GBC and its associated DFS. A retrospective study conducted in Japan established that a TTT of up to 90 days did not affect survival in patients with clinical stage II/III gastric cancer.^38^ Moreover, in the case of colon cancer, the delayed TTT within 40 days did not appear to have a significant effect on OS in stage I‐III colon cancer.^39^ However, no study has been conducted to date to examine the influencing factors involving TTT on both OS and DFS in patients with T1b/T2 GBC. Meanwhile, it remained unknown whether the laparoscopic or open approach should be used in the case of different TTT.

Our study revealed the association of several potential influencing factors, including TTT, with survival outcomes, surgery‐related outcomes, and selection of surgical approaches for GBC, which remained unclear previously. Meanwhile, referrals were identified as the main driver for the highly selective group of T1b/T2 GBC patients. Although GBC contributed the majority of biliary tract cancers, it is still a relatively uncommon malignancy.^40^ With the accumulation of time and the increasing incidence, the number of GBC cases would continue to grow, verifying and updating our findings potentially. Notably, it is essential to timely determine the relationship between TTT and prognosis and even to shorten TTT for GBC patients as targeted therapy and immunotherapy popularize in present and future.^10^

This study has several limitations. First, the retrospective study was limited to an Asian population with a small sample size, and we would include more patients worldwide in future studies. Second, we focused on limited tumor stages, so the findings cannot be generalized to patients with more advanced GBC or other types of cancer. Moreover, the study examined the effect of TTTs up to 28 days (the maximal TTT) based on the cutoff of TTT of 7 days, and a large multicenter study should be conducted to determine the association between longer TTT (>28 days) and survival outcomes based on the adjusted cutoff for both GBC and IGBC patients. Meanwhile, other factors would be potentially identified as the drivers of TTT in future studies such as smoking history, comorbidities, preoperative imaging examination and intraoperative histopathology for GBC, gallstones, and multidisciplinary assessment. Finally, GBC treatment varies from surgery to chemotherapy, radiotherapy, targeted therapy, and immunotherapy,^41^ whereas the study merely examined the effect of TTT for surgery.

In conclusion, we demonstrated that not having referrals, fewer positive LNs, and well differentiation were all associated with better OS, whereas fewer positive LNs were associated with better DFS. As a potential influencing factor, the prolonged TTT (up to 28 days) induced by referrals had no adverse effect on survival and surgery‐related outcomes in T1b/T2 GBC patients. Additionally, surgeons should prioritize surgical approaches (laparoscopic or open) over TTT. These findings would also be used to help patients eliminate the fear of postponed surgery for T1b/T2 GBC and to inform surgical approaches selection.

## AUTHOR CONTRIBUTIONS

**Jiasheng Cao:** Conceptualization (equal); data curation (lead); formal analysis (lead); investigation (equal); methodology (equal); writing – original draft (lead); writing – review and editing (equal). **Jiafei Yan:** Conceptualization (equal); data curation (equal). **Jiahao Hu:** Conceptualization (equal); data curation (equal). **Bin Zhang:** Formal analysis (equal). **Win Topatana:** Formal analysis (equal). **Shijie Li:** Writing – original draft (equal). **Tianen Chen:** Formal analysis (supporting). **Sarun Juengpanich:** Writing – original draft (equal). **Ziyi Lu:** Writing – original draft (supporting). **Shuyou Peng:** Writing – review and editing (equal). **Xiujun Cai:** Formal analysis (lead); writing – review and editing (lead). **Mingyu Chen:** Conceptualization (lead); supervision (lead); writing – review and editing (lead).

## FUNDING INFORMATION

This work was supported by the National Natural Science Foundation of China (No. 82202873), the Natural Science Foundation of Zhejiang Province (No. LQ22H160003 and No. LQ23H160036), and the Fundamental Research Funds for the Central Universities (No. 2022QZJH52).

## CONFLICT OF INTEREST STATEMENT

The authors deny any conflict of interest.

## CLINICAL TRIAL REGISTRATION NUMBER

The study has been registered in the Chinese Clinical Trial Registry (ChiCTR2200057050).

## Supporting information


Data S1.

## Data Availability

All original data are available upon reasonable request to the corresponding authors.
