# Supplementary material for: Estimating the influencing factors for T1b/T2 gallbladder cancer on survival and surgical approaches selection
Source: Cancer Med. 2023 Jun 27;12(16):16744–55. doi: 10.1002/cam4.6297 (PMC10501227; doi:10.1002/cam4.6297)
Supplement: Supplementary file 1 — Data S1. [file CAM4-12-16744-s001.docx]

**Supplementary Materials**

**Estimating the Influencing Factors for T1b/T2 Gallbladder Cancer on Survival and Surgical Approaches Selection**

Jiasheng Cao, MD^1,2†^, Jiafei Yan, MD^1,2†^, Jiahao Hu, MD^1,2†^, Bin Zhang, MD^1,2^, Win Topatana, MD^2^, Shijie Li, MD^1,2^, Tianen Chen, MD^1,2^, Sarun Jeungpanich, MD^2^, Ziyi Lu, MD^2^, Shuyou Peng, MD, PhD^1,2,3^, Xiujun Cai, MD, PhD^1,2*^, Mingyu Chen, MD, PhD^1,2*^

^1^ Department of General Surgery, Sir Run-Run Shaw Hospital, Zhejiang University, Hangzhou 310016, Zhejiang Province, China.

^2^ Zhejiang University School of Medicine, Zhejiang University, Hangzhou 310058, Zhejiang Province, China.

^3^ Department of General Surgery, the Second Affiliated Hospital, Zhejiang University, Hangzhou 310009, Zhejiang Province, China.

**^†^ Contributed equally.**

**^*^ Corresponding authors:**

**Xiujun Cai**, MD, PhD, FACS, FRCS, IHPBA member, ELSA member, Department of General Surgery, Sir Run-Run Shaw Hospital, Zhejiang University, No.3 East Qingchun Road, Hangzhou 310016, Zhejiang Province, China. Email: srrsh_cxj@zju.edu.cn, China, Tel: 86-571-86006617, Fax: 86-571-86044817.

**Mingyu Chen**, MD, PhD, EASL member, ESMO member, Department of General Surgery, Sir Run-Run Shaw Hospital, Zhejiang University, No.3 East Qingchun Road, Hangzhou 310016, Zhejiang Province, China. Email: mychen@zju.edu.cn, Tel: 86-571-86006617, Fax: 86-571-86044817.

**This file includes: Figure S1 - S4 and Table S1 - S3**

**Figure S1**

**
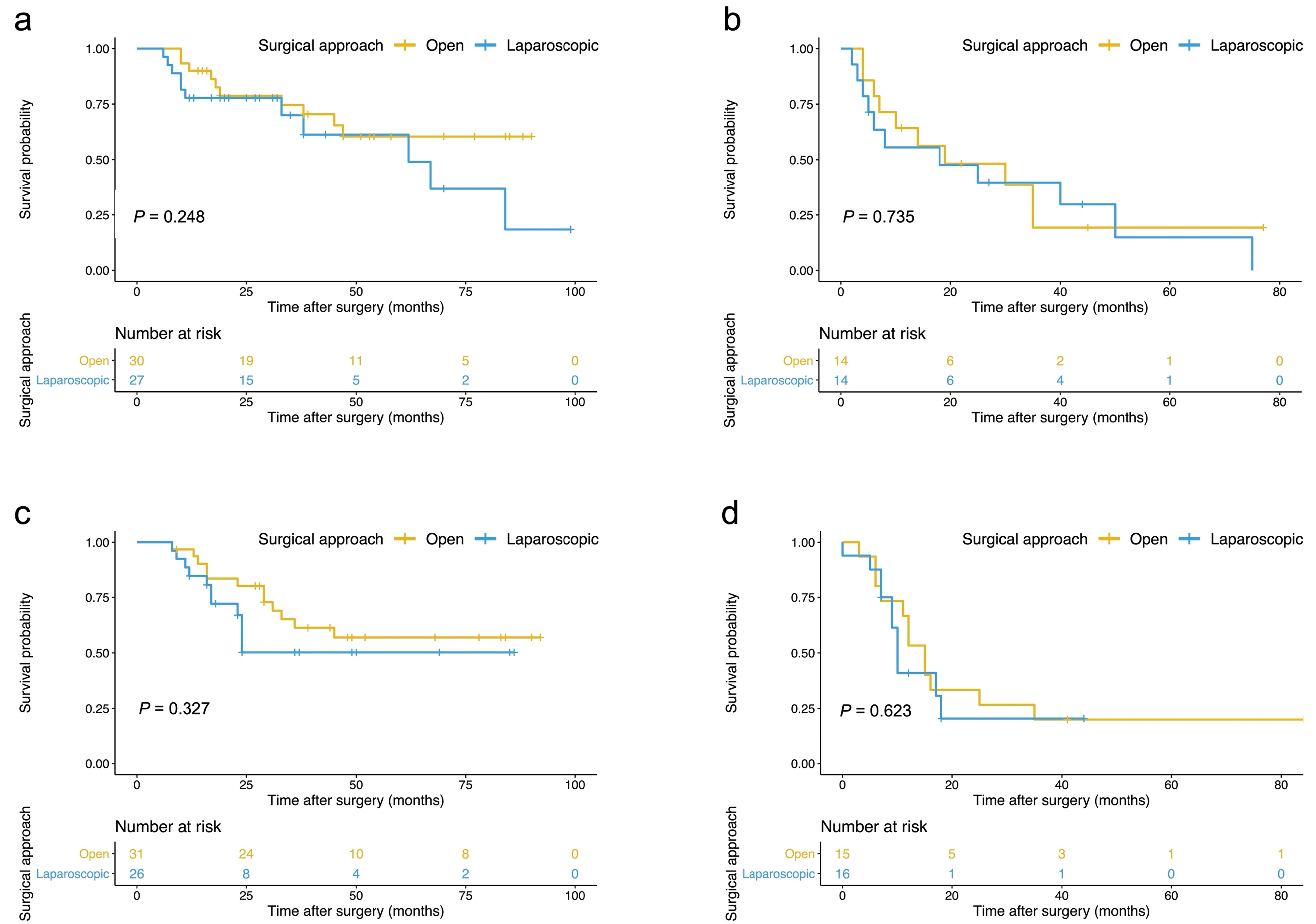
**

**Figure S1:** Comparisons of survival outcomes of different surgical approaches based on TTT in T1b/T2 GBC patients. The difference of (a) overall survival (HR, 1.665; 95% CI, 0.701 - 3.956; *P* = 0.248) and (b) disease-free survival (HR, 1.161; 95% CI, 0.489 - 2.754; *P* = 0.735) between patients undergoing laparoscopic and open approach in short TTT group. The difference of (c) overall survival (HR, 1.513; 95% CI, 0.662 - 3.460; *P* = 0.327) and (d) disease-free survival (HR, 1.234; 95% CI, 0.534 - 2.849; *P* = 0.623) between patients undergoing laparoscopic and open approach in long TTT group. TTT, Time to treatment; GBC, Gallbladder cancer; HR, Hazard ratio; CI, Confidence interval.

**Figure S2**

**
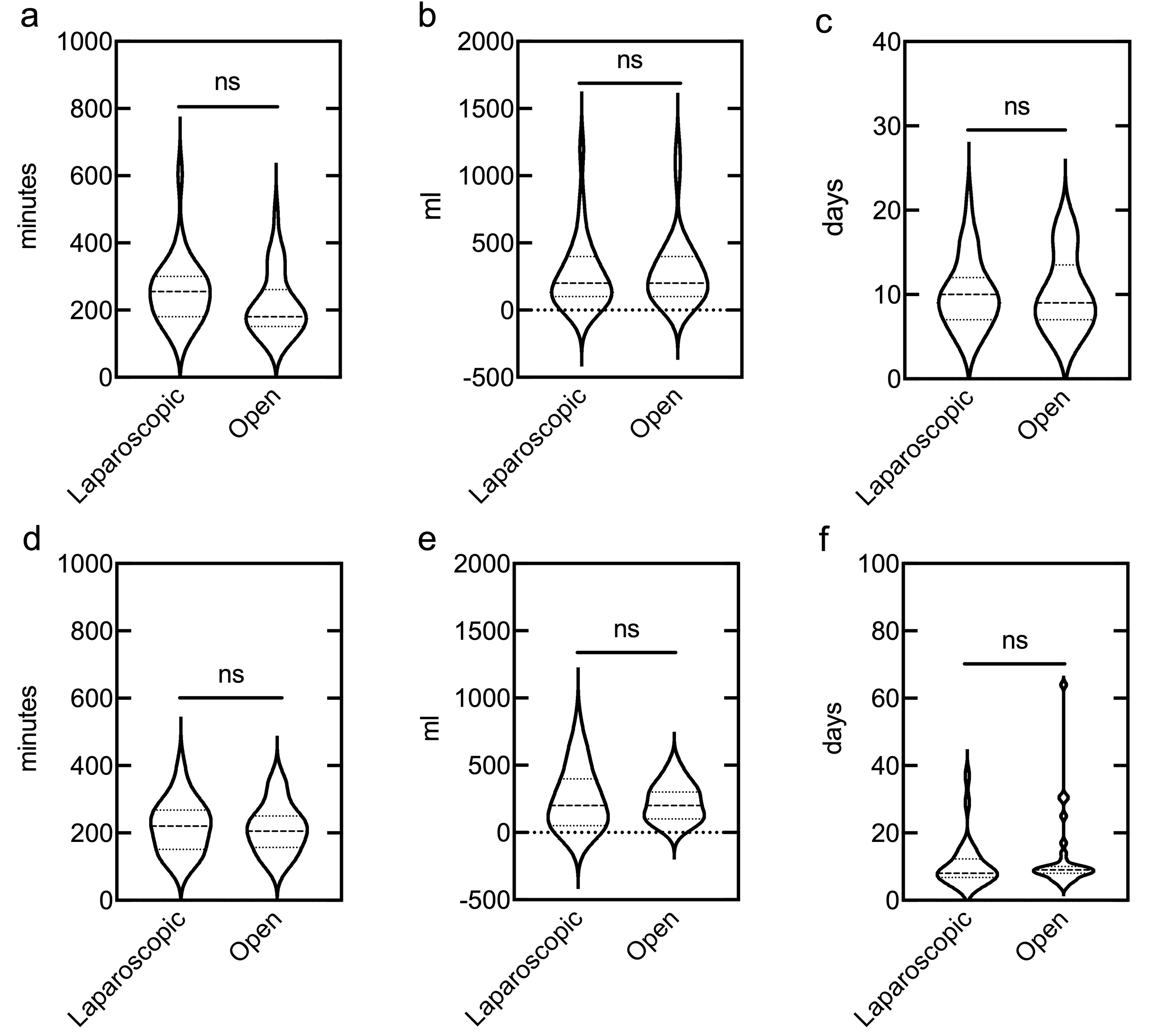
**

**Figure S2:** Comparisons of surgery-related outcomes of different surgical approaches based on TTT in T1b/T2 GBC patients. The difference of (a) operation time (*P* = 0.056), (b) intraoperative blood loss (*P* = 0.472), and (c) postoperative hospital length of stay (*P* = 0.895) between patients undergoing laparoscopic and open approach in short TTT group. The difference of (d) operation time (*P* = 0.747), (e) intraoperative blood loss (*P* = 0.838), and (f) postoperative hospital length of stay (*P* = 0.514) between patients undergoing laparoscopic and open approach in short TTT group. TTT, Time to treatment; GBC, Gallbladder cancer; NS, Not significant.

**Figure S3**

**
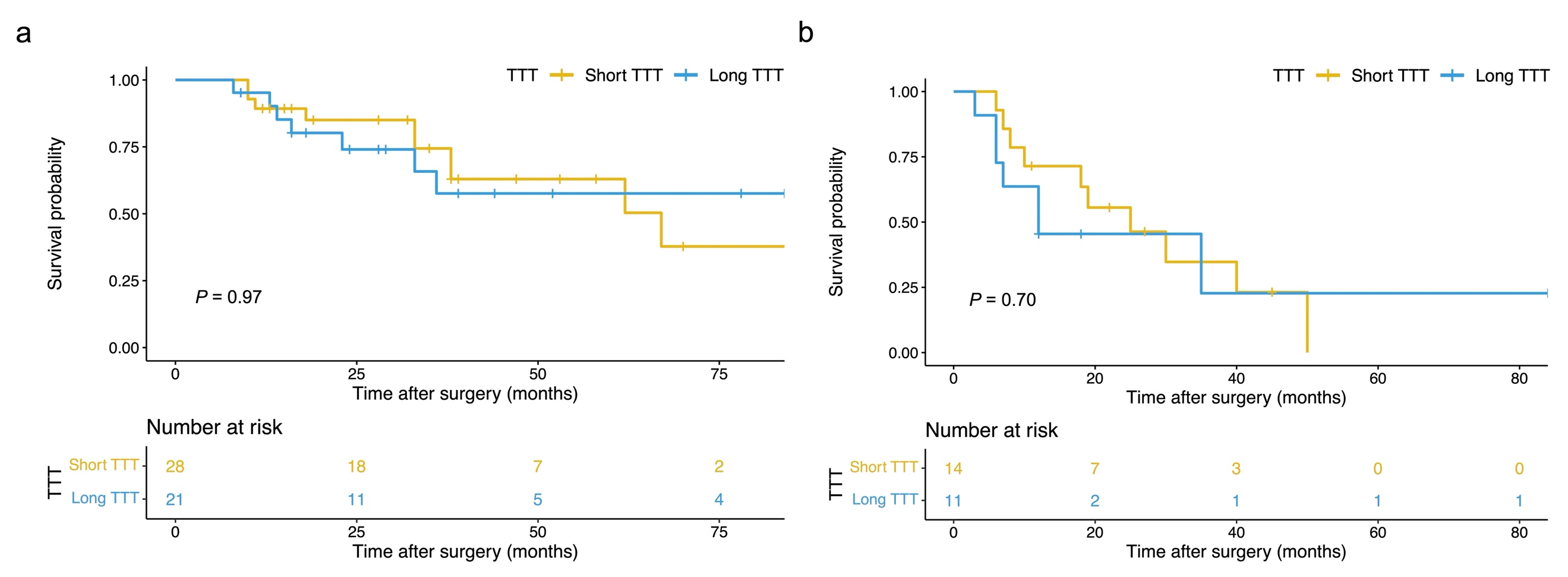
**

**Figure S3:** Comparisons of survival outcomes of T1b/T2 IGBC. The difference of (a) overall survival and (b) disease-free survival between short TTT group and long TTT group. IGBC, Incidental gallbladder cancer; TTT, Time to treatment.

**Figure S4**

**
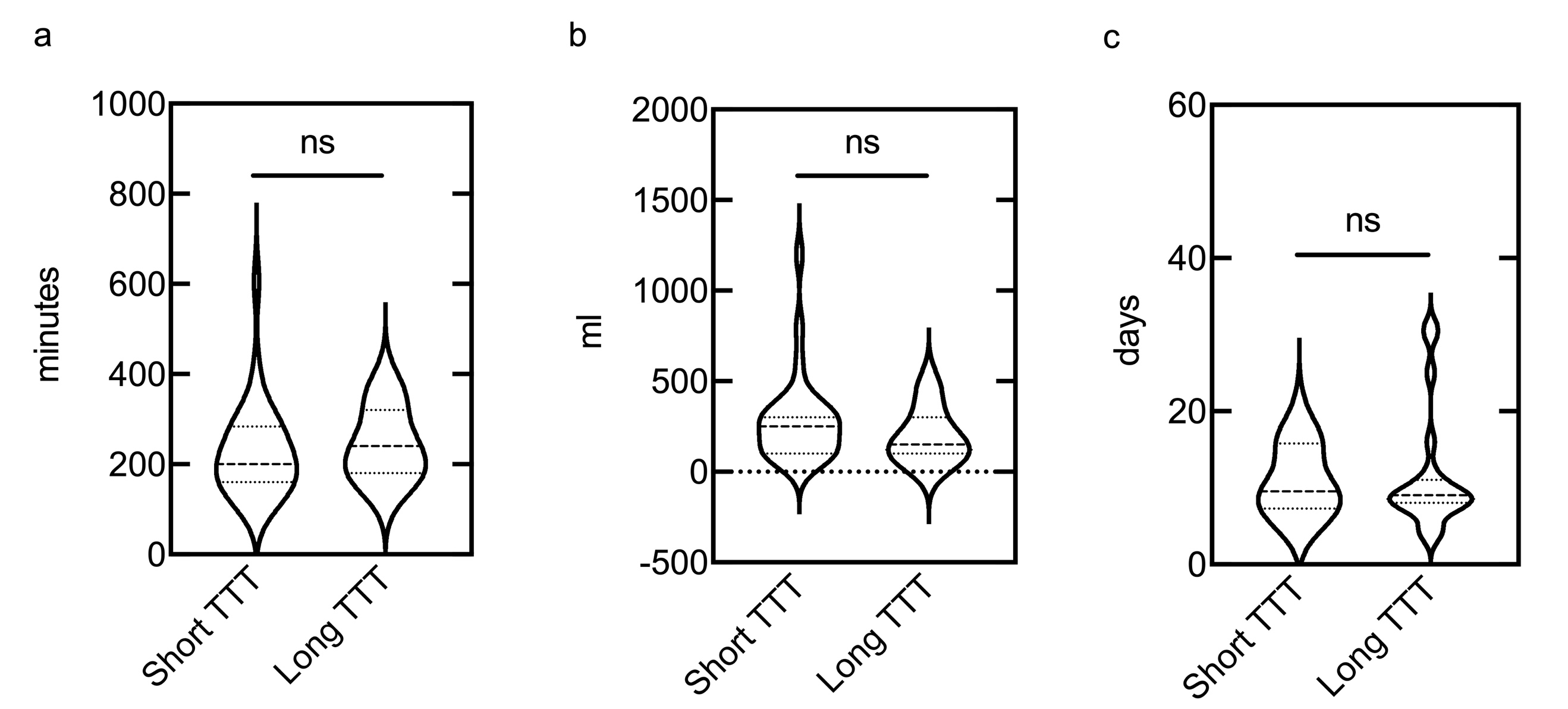
**

**Figure S4:** Comparisons of surgery-related outcomes of T1b/T2 IGBC patients between two groups. The difference of (a) operation time (*P* = 0.322), (b) intraoperative blood loss (*P* = 0.176), and (c) postoperative hospital length of stay (*P* = 0.549) between short TTT group and long TTT group. IGBC, Incidental gallbladder cancer; TTT, Time to treatment.

| **Table S1. Patient characteristics of different surgical approach groups with short TTT and long TTT.** | | | | | | | |
| --- | --- | --- | --- | --- | --- | --- | --- |
| **Variables** | **Short TTT (≤ 7 days) (n = 57) n (%) or median [range]** | | |  | **Long TTT (> 7 days) (n = 57) n (%) or median [range]** | | |
|  | **Laparoscopic approach (n = 27)** | **Open approach (n = 30)** | ***P* value** |  | **Laparoscopic approach (n = 26)** | **Open approach (n = 31)** | ***P* value** |
| Age (y) | 59 [48 - 75] | 67 [45 -78] | 0.113 |  | 61.5 [49 - 77] | 60 [39 - 79] | 0.308 |
| Sex |  |  | 0.506 |  |  |  | **0.014*** |
| Male | 6 (22.2) | 9 (30.0) |  |  | 12 (46.2) | 5 (16.1) |  |
| Female | 21 (77.8) | 21 (70.0) |  |  | 14 (53.8) | 26 (83.9) |  |
| BMI ≥ 25 kg/m2 | 7 (25.9) | 15 (50.0) | 0.062 |  | 7 (26.9) | 12 (38.7) | 0.347 |
| Smoking history | 0 (0) | 3 (10.0) | 0.091 |  | 2 (7.7) | 2 (6.5) | 0.855 |
| Comorbidities | 8 (29.6) | 15 (50.0) | 0.118 |  | 11 (42.3) | 11 (35.5) | 0.598 |
| Preoperative suspicion or intraoperative diagnosis |  |  | 0.696 |  |  |  | 0.514 |
| Imaging examination (Ultrasonography/CT/MRI/PET-CT) | 13 (48.1) | 16 (53.3) |  |  | 12 (46.2) | 17 (54.8) |  |
| Histopathology | 14 (51.9) | 14 (46.7) |  |  | 14 (53.8) | 14 (45.2) |  |
| Referrals | 5 (18.5) | 5 (16.7) | 0.854 |  | 18 (69.2) | 16 (51.6) | 0.177 |
| Gallbladder stones | 9 (33.3) | 17 (56.7) | 0.077 |  | 9 (34.6) | 12 (38.7) | 0.750 |
| Preoperative CA19-9 (≤ 37 U/ml) | 21 (77.8) | 24 (80.0) | 0.837 |  | 18 (69.2) | 22 (71.0) | 0.886 |
| Preoperative CEA (≤ 5 ng/mL) | 24 (88.9) | 28 (93.3) | 0.554 |  | 20 (76.9) | 27 (87.1) | 0.314 |
| Tumor size (cm) |  |  | 0.063 |  |  |  | 0.451 |
| ≤ 1 | 4 (14.8) | 11 (36.7) |  |  | 2 (7.7) | 5 (16.1) |  |
| 1 - 3 | 17 (63.0) | 10 (33.3) |  |  | 13 (50.0) | 17 (54.8) |  |
| > 3 | 6 (22.2) | 9 (30.0) |  |  | 11 (42.3) | 9 (29.0) |  |
| T stage |  |  | 0.913 |  |  |  | 0.391 |
| T1b | 2 (7.4) | 2 (6.7) |  |  | 1 (3.8) | 3 (9.7) |  |
| T2 | 25 (92.6) | 28 (93.3) |  |  | 25 (96.2) | 28 (90.3) |  |
| Hepatectomy type |  |  | 0.617 |  |  |  | 0.103 |
| Wedge resection | 26 (96.3) | 28 (93.3) |  |  | 26 (100.0) | 28 (90.3) |  |
| Segment IVb/V resection | 1 (3.7) | 2 (6.7) |  |  | 0 (0) | 3 (9.7) |  |
| Total harvested LNs | 6 [1 - 14] | 8 [1 - 20] | 0.671 |  | 1 [6 -16] | 9 [1 - 42] | **0.046*** |
| Positive LNs | 0 [0 - 4] | 0 [0 - 5] | 0.694 |  | 0 [0 - 6] | 0 [0 - 5] | 0.612 |
| Tumor differentiation |  |  | 0.428 |  |  |  | 0.157 |
| Well | 13 (48.1) | 15 (50.0) |  |  | 9 (34.6) | 17 (54.8) |  |
| Moderate | 7 (25.9) | 4 (13.3) |  |  | 5 (19.2) | 7 (22.6) |  |
| Poor | 7 (25.9) | 11 (36.7) |  |  | 12 (46.2) | 7 (22.6) |  |
| Postoperative adjuvant treatment |  |  | 0.537 |  |  |  | 0.179 |
| Chemotherapy | 4 (14.8) | 2 (6.7) |  |  | 4 (15.4) | 1 (3.2) |  |
| Radiotherapy | 0 (0) | 0 (0) |  |  | 2 (7.7) | 0 (0) |  |
| Chemoradiotherapy | 7 (25.9) | 5 (16.7) |  |  | 4 (15.4) | 5 (16.1) |  |
| Immunotherapy | 0 (0) | 0 (0) |  |  | 1 (3.8) | 0 (0) |  |
| Targeted therapy | 0 (0) | 0 (0) |  |  | 0 (0) | 0 (0) |  |
| Supportive care | 15 (55.6) | 22 (73.3) |  |  | 15 (57.7) | 24 (74.4) |  |
| Traditional medicine | 1 (3.7) | 1 (3.3) |  |  | 0 (0) | 1 (3.2) |  |
| TTT, Time to treatment; BMI, Body mass index; CT, Computed tomography; MRI, Magnetic resonance imaging; PET-CT, Positron emission tomography-computed tomography; CA19-9, Carbohydrate antigen 19-9; CEA, Carcinoembryonic antigen; LNs, Lymph nodes; NA, Not available.  ***** *P* < 0.05 **Note:** T stage was based on the 8^th^ American Joint Committee on Cancer Staging Manual. | | | | | | | |

| **Table S2. Patient characteristics of IGBC patients.** | |
| --- | --- |
| **Variables** | **Study cohort (n = 49) n (%) or median [range]** |
| TTT (days) | 7 [0 - 26] |
| Gallbladder stones | 26 (53.1) |
| Gallbladder polypoid lesions | 11 (22.4) |
| Reasons for referral |  |
| Abdominal pain | 33 (67.3) |
| Abdominal distension | 1 (2.0) |
| Examination for gallbladder stones | 7 (14.3) |
| Examination for gallbladder polypoid lesions | 8 (16.3) |
| Symptoms |  |
| Abdominal pain | 32 (65.3) |
| Abdominal distension | 2 (4.1) |
| None | 15 (30.6) |
| Time interval from symptom onset to operation |  |
| < 1w | 2 (4.1) |
| 1w ~ 1m | 12 (25.0) |
| 1m ~ 1y | 17 (35.4) |
| 1y ~ 5y | 6 (12.5) |
| 5y ~ 10y | 6 (12.5) |
| > 10y | 6 (12.5) |
| T stage |  |
| T1b | 5 (10.2) |
| T2 | 44 (89.8) |
| IGBC, Incidental gallbladder cancer; TTT, Time to treatment. w, week; m, month; y, year. | |

| **Table S3. Patient characteristics of IGBC patients based on different TTT.** | | | |
| --- | --- | --- | --- |
| **Variables** | **Short TTT (≤ 7 days)**  **(n = 28) n (%) or median [range]** | **Long TTT (> 7 days)**  **(n = 21) n (%) or median [range]** | ***P* value** |
| Age (y) | 63.5 [46 - 76] | 61 [39 - 79] | 0.836 |
| Sex |  |  | 0.253 |
| Male | 11(39.3) | 5 (23.8) |  |
| Female | 17 (60.7) | 16 (76.2) |  |
| BMI ≥ 25 kg/m2 | 12 (42.9) | 6 (28.6) | 0.305 |
| Smoking history | 2 (7.1) | 1 (4.8) | 0.731 |
| Comorbidities | 13 (46.4) | 5 (23.8) | 0.169 |
| Referrals | 1 (3.6) | 8 (38.1) | **0.002*** |
| Gallbladder stones | 14 (50.0) | 12 (57.1) | 0.620 |
| Preoperative CA19-9 (≤ 37 U/ml) | 24 (85.7) | 17 (81.0) | 0.655 |
| Preoperative CEA (≤ 5 ng/mL) | 27 (96.4) | 19 (90.5) | 0.390 |
| Tumor size (cm) |  |  | 0.341 |
| ≤ 1 | 11 (39.3) | 5 (23.8) |  |
| 1 - 3 | 14 (50.0) | 11 (52.4) |  |
| > 3 | 3 (10.7) | 5 (23.8) |  |
| T stage |  |  | 0.414 |
| T1b | 2 (7.1) | 3 (14.3) |  |
| T2 | 26 (92.9) | 18 (85.7) |  |
| Surgical approach |  |  | **0.026*** |
| Laparoscopic approach | 14 (50.0) | 17 (81.0) |  |
| Open approach | 14 (50.0) | 4 (19.0) |  |
| Hepatectomy type |  |  | 0.243 |
| Wedge resection | 28 (100.0) | 20 (95.2) |  |
| Segment IVb/V resection | 0 (0) | 1 (4.8) |  |
| Total harvested LNs | 6.5 [1 - 15] | 8 [1 - 25] | 0.421 |
| Positive LNs | 0 [0 - 1] | 0 [0 - 5] | 0.221 |
| Tumor differentiation |  |  | 0.192 |
| Well | 10 (35.7) | 13 (61.9) |  |
| Moderate | 9 (32.1) | 4 (19.0) |  |
| Poor | 9 (32.1) | 4 (19.0) |  |
| IGBC, Incidental gallbladder cancer; BMI, Body mass index; CA19-9, Carbohydrate antigen 19-9;  CEA, Carcinoembryonic antigen; LNs, lymph nodes.  ***** *P* < 0.05 **Note:** T stage was based on the eighth American Joint Committee on Cancer Staging Manual. | | | |
